# Current Status and Future Targeted Therapy in Adrenocortical Cancer

**DOI:** 10.3389/fendo.2021.613248

**Published:** 2021-03-01

**Authors:** George Alyateem, Naris Nilubol

**Affiliations:** Surgical Oncology Program, Center for Cancer Research, National Cancer Institute, National Institutes of Health, Bethesda, MD, United States

**Keywords:** adrenocortical, cancer, drug discovery, clinical trial, genomics, molecular profiling, targeted therapy

## Abstract

Adrenocortical carcinoma (ACC) is a rare malignancy with a poor prognosis. The current treatment standards include complete surgical resection for localized resectable disease and systemic therapy with mitotane alone or in combination with etoposide, doxorubicin, and cisplatin in patients with advanced ACC. However, the efficacy of systemic therapy in ACC is very limited, with high rates of toxicities. The understanding of altered molecular pathways is critically important to identify effective treatment options that currently do not exist. In this review, we discuss the results of recent advanced in molecular profiling of ACC with the focus on dysregulated pathways from various genomic and epigenetic dysregulation. We discuss the potential translational therapeutic implication of molecular alterations. In addition, we review and summarize the results of recent clinical trials and ongoing trials.

## Introduction

Adrenocortical carcinoma (ACC) is a rare malignancy arising from the adrenal cortex, with an annual worldwide incidence of 0.5–2 individuals per million population (1, 2). The median age at diagnosis is 55 years, though the incidence follows a bimodal pattern of age distribution, with peaks before the age of five and between the fourth and fifth decades of life (3, 4). Although the disease aggressiveness varies, the prognosis of patients with ACC is generally poor with a median overall survival of approximately 4 years (2), partly due to the late stage at presentation. Only one-third of patients with ACC in the US presented at TNM stage I or II (5). In a French study, five-year overall survival was 66, 58, 24, and 0% for stages 1, 2, 3, and 4 ACC, respectively (6). Alternatively, the prognosis of patients with ACC can be categorized by the extent of the disease. Five-year overall survival of patients with localized disease (limited to the adrenal gland), regionalized disease (locally advanced), and those with distant metastasis was 60–80%, 35–50%, and 0–28%, respectively (7). Cushing’s syndrome is observed in 50–60% of patients with ACC. Hyperandrogenism is seen in 20–30% of female patients, with a small number of those patients having estrogen and/or mineralocorticoid excess. Primary aldosteronism can also be seen in only 2.5% of patients with ACC (3). Patients may also experience weight loss, fatigue, night sweats, or fever (8, 9). Prognosis also differs with regard to age, extent of surgical resection (R0, R1, R2), mitotic rate, and hormone secretion. A 10-year follow-up study of 180 patients that underwent resection of adrenocortical carcinoma clearly stratify this data (10). Data was stratified into cohorts by amount of time patients were alive after surgery (e.g., patients alive <2 years, alive 2–5 years, alive 5–10 years, alive >10 years). Of the 37 patients alive 5–10 years, 78.1% had an R0 resection, 21.9% had an R1 resection, and 0 patients were alive that had an R2 resection. Of these same patients, 48.5% had a non-secreting tumor, 24.2% had a cortisol-secreting tumor, and 18.2% had a non-cortisol secreting tumor. Neither age nor high mitotic rate did not seem to affect overall survival for any patient cohort in their review.

**Figure 1:**
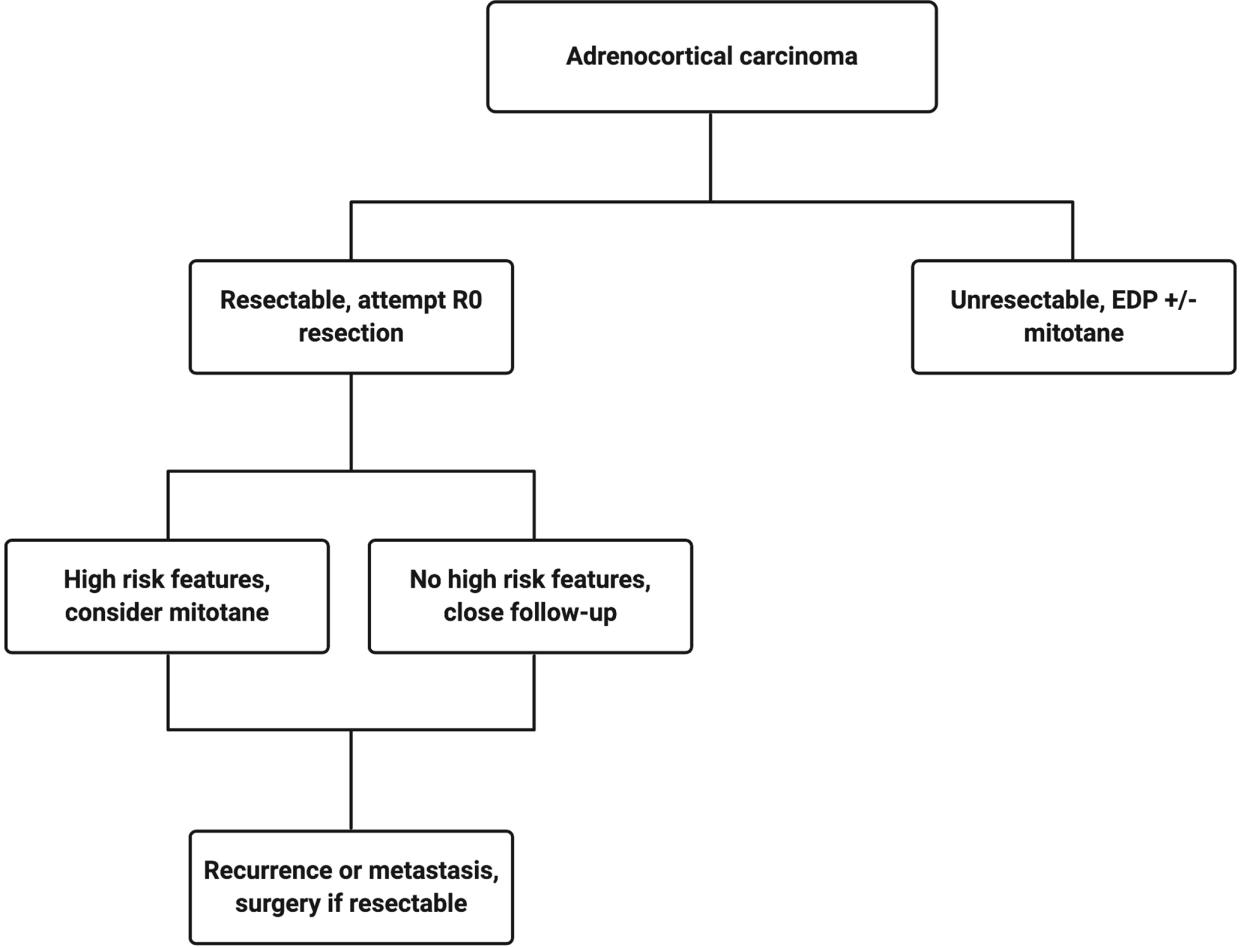
Treatment algorithm for a patient with confirmed adrenocortical carcinoma. Created with BioRender.com.

**Figure 2:**
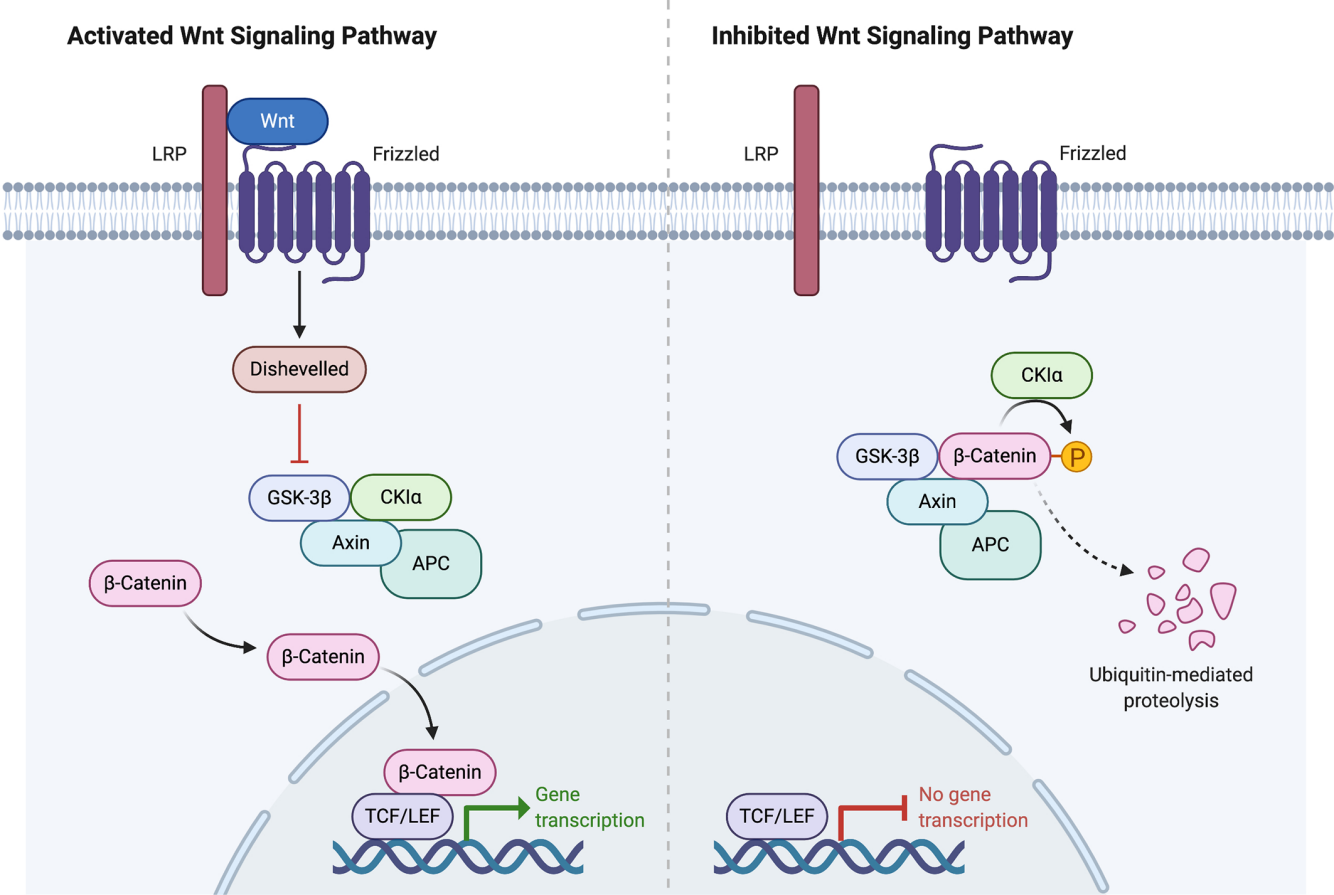
Wnt signaling pathway activation and inhibition. Created with BioRender.com.

The current treatment scheme is patients with ACC is summarized in **Figure 1**. Patients with localized and regionalized ACC have a potential for cure with complete surgical resection (7). Yet, even with an R0 resection, 50–80% of patients develop recurrent or metastatic disease (11, 12). The role of surgery in patients with recurrent or metastatic disease remains a topic of debate. Some recent studies demonstrated a modest survival benefit in *selected* patients that underwent surgery for a recurrent disease if the disease-free interval was greater than 12 months (13, 14). Because of the lack of effective systemic treatments, patients with resectable, recurrent and/or metastatic ACC should be evaluated for surgery when the disease progression is not rapid, such as those who do not develop new metastatic lesions within 6 months of diagnosis. Patient selection should be based on a thorough discussion of surgical risks, the benefits of achieving “no evidence of disease” status, and the risk and time of recurrence in the absence of level 1 data (**Figure 2**). The *selected* patients with advanced ACC may benefit from metastasectomy (14–16). Since the efficacy and the options of systemic treatment are limited, an aggressive surgical approach may be recommended in patients with advanced ACC that follows a relatively more indolent course.

Mitotane is the only FDA-approved systemic therapy in ACC. It is an adrenolytic agent derived from the insecticide dichlorodiphenyltrichloroethane (DDT). Mitotane has been the standard treatment for patients with advanced-stage ACC for decades to control tumor growth and hypercortisolemia. However, the response rates are only about 30% and the systemic toxicities make it difficult for patients to tolerate due to a narrow therapeutic window (17–19). In patients with advanced ACC who are not surgical candidates, mitotane combined with platinum-based chemotherapy is recommended as first-line treatment. The recommendation is based on data from the FIRM-ACT randomized clinical trial that compared mitotane plus streptozocin with mitotane plus etoposide, doxorubicin, and cisplatin (EDP-M). Patients who received EDP-M as the first-line therapy had a significantly higher rate of tumor response (23.2% vs. 9.2%, p < 0.001), but only translated to 3 months longer median progression-free survival, compared to the streptozocin group (5.0 vs. 2.1 months, p < 0.001). At five years, 15–20% of patients in the EDP-M group remained alive, compared to 5–10% of patients in the mitotane plus streptozocin cohort (20).

Patients with ACC mostly present sporadically; however, several hereditary syndromes are associated with the development of ACC. The insight into ACC carcinogenesis from these syndromes can be useful in identifying new treatments. These inherited syndromes include Li-Fraumeni, Beckwith-Wiedemann, multiple endocrine neoplasia type 1 (MEN1), Lynch, familial adenomatous polyposis syndromes, and Carney complex. Li-Fraumeni syndrome is inherited in the autosomal dominant pattern and is associated with inactivating pathogenic variant in *TP53* on chromosome 17p13.1. In children, germline *TP53* variants could be detected in 50–88% of patients with ACC (21, 22). In adults with ACC, germline pathogenic variants in *TP53* are seen in 4–6% of patients (23, 24). Li-Fraumeni syndrome also confers susceptibility to breast carcinoma, soft tissue sarcoma, brain tumors, osteosarcoma, and leukemia (25). In Beckwith-Wiedemann syndrome, ACC tumors exhibit pathogenic variations or deletions of imprinted genes on chromosome 11p15. Patients with Beckwith-Wiedemann syndrome are also susceptible to developing congenital abnormalities (omphalocele, macroglossia, macrosomia, hemihypertrophy) or Wilm’s tumor. Patients with Lynch syndrome have a lifetime risk of colorectal cancer of 10–47%, depending on the mismatch repair gene that is mutated. Lynch syndrome has also been linked to increased rates of other cancers, including adrenocortical carcinoma, pancreatic, prostate, breast, and cervical cancer. Patients with MEN1 syndrome have inactivating pathogenic variants of the *MEN1* gene that increase susceptibility to parathyroid tumors, pituitary tumors, pancreatic neuroendocrine tumors, and unilateral or bilateral adrenal tumors, including adrenocortical carcinoma. Familial adenomatous polyposis occurs secondary to a pathogenic germline variation in the *APC* gene and is associated with the development of multiple adenomatous polyps and cancer of the colon and rectum, along with extracolonic manifestations that include adrenocortical carcinoma (26–30). Lastly, Carney complex is a multiple neoplasia syndrome characterized by a pathogenic variant of the *PRKAR1A* gene resulting in spotty skin pigmentation, myxomas, pasmmomatous melanotic schwannomas, and endocrine tumors. A case report (31) and genetic analysis of a large family with Carney complex (32) point to an association between Carney complex and ACC.

Because of the lack of effective systemic treatment options, there is an urgent need for effective therapies in the management of adrenocortical carcinoma. A better understanding of the molecular drivers that contribute to ACC development is critical. In this review, we focus on the molecular alterations of ACC and the potential therapeutic implications.

## Genetic Alterations

Two important studies that comprehensively analyzed ACC samples were from the European Network for the Study of Adrenal Tumors (ENSAT) (33) and The Cancer Genome Atlas (34) cohorts. These two studies integratively analyzed multiple molecular and genomic platforms. They discovered and confirmed several important molecular alterations in ACC tumorigenesis and progression. The publicly-available databases from both studies containing somatic mutations, DNA methylation, mRNA expression, miRNA expression, and proteomics in ACC have been an invaluable resource for researchers. The following sections will briefly summarize the molecular alterations associated with ACC.

### Whole Exome Sequencing data

Assie et al. (ENSAT) performed the whole-exome sequencing (WES) on 45 ACC tumors and found 3,153 somatic mutations, 1,881 of which occurred in two tumors with a hypermutation phenotype (33). Zheng et al. (TCGA) performed WES on 91 tumors and found 8,841 mutations, 3,427 of which were found in two tumors with an ultramutator phenotype (34). The ultramutator phenotype was excluded from subsequent whole-exome analyses. Compared to other cancers with the median tumor mutation of 3.6 mutations per megabase (35), the tumor mutation burden in ACC is considered relatively low. ACC from ENSAT cohort displayed a mean somatic mutation rate of 0.6 mutations per megabase, whereas those from TCGA cohort showed a median somatic density of 0.9 mutations per megabase. However, both studies showed a linear relationship between the number of mutations in an ACC tumor and worse 5-year overall survival, higher Weiss score, and higher ENSAT stage (33, 34).

### Somatic Mutations

Copy number alterations were profiled *via* single-nucleotide polymorphism (SNP) array. In the ENSAT study, 16/22 autosomes showed the loss of heterozygosity in greater than 30% of cases. As previously reported (36), the *IGF2* locus showed frequent loss of heterozygosity, and was seen in 82% of tumors in the ENSAT study. The TGCA study reported that *IGF2* expression was unanimously high in 67/78 tumors, and the expression was independent of ACC classification (33, 34).

In the ENSAT study, high-level amplifications were seen in *TERT* and *CDK4*. Homozygous deletions were noted in *CDKN2A*, *RB1*, *ZNRF3*, 3q13.1, 4q34.3, and around a long noncoding RNA *LINC00290*. Chromosomal analysis revealed hypodiploidy in 33% of tumors and polyploidy in 43% of tumors. The most frequently altered gene was *ZNRF3*, with changes seen in 21% of ACC tumors in the ENSAT cohort. *ZNRF3* encodes a cell surface membrane E3 ubiquitin ligase that is a negative feedback regulator of the Wnt/β-catenin signaling pathway by promoting the degradation of the LRP6 and Frizzled receptors (**Figure 2**). Homozygous deletions of *ZNRF3* were seen in 19 tumors, and somatic mutations were noted in 7 more tumors (33, 34).

In the TCGA study, recurrent focal amplifications were similarly noted in *TERT* and *CDK4*, but their analysis added *TERF2* (Telomeric Repeat-Binding Factor2) and *CCNE1* (Cyclin E1) to the list developed previously by the ENSAT study. Similar to ENSAT cohorts, TCGA study found deletions in *CDKN2A*, *RB1*, *ZNRF3*, and around *LINC00290* in ACC. ABSOLUTE algorithm (37) was used to determine tumor purity, ploidy, and give insight into whole-genome doubling. Hypodiploidy was noted in 31% of tumors (which was higher than 11 other tumor types). The whole-genome doubling analysis led to an evaluation of telomere regulation. *TERT* expression was significantly higher in tumors that underwent the whole-genome doubling, leading the authors to postulate that the relationship between the whole-genome doubling and *TERT* expression suggests the important role *TERT* plays in maintaining telomere length in ACC (33, 34).

MutSigCV (38) is a robust analytical methodology to identify gene mutations associated with cancers. This method overcomes the mutational heterogeneity. Using this method, *CTNNB1*, *TP53*, *DAXX*, *MEN1*, *PRKAR1A*, *RPL22* were all identified collectively between the ENSAT and TCGA cohorts. Of 122 ACC tumors evaluated in the ENSAT study, nine genes displayed damaging mutations, homozygous deletions, or high-level amplifications in ≥ 5% of ACCs: *ZNRF3*, *CTNNB1*, *TP53*, *CDKN2A*, *RB1*, *MEN1*, *DAXX*, *MED12*, and *TERT*. The TCGA group analyzed their data *via* the Cancer Gene Consensus and also noted that *NF1* and *MLL4* were mutated in more than 5% of the cohort (33, 34).

Alterations in *CTNNB1*, a gene that encodes the β-catenin protein, and *ZNRF3* were noted to be mutually exclusive. β-catenin targets were activated *via* transcription in tumors with altered *ZNRF3* were seen, but this activation was weaker than in *CTNNB1-*mutated tumors. Alterations of *ZNRF3*, *CTNNB1*, *APC*, and *MEN1* resulted in modification of the Wnt/β-catenin pathway in 41% of TCGA tumors, and 39% of ENSAT tumors. Alterations in the p53-Rb pathway were noted in 33% of tumors in the ESNAT study. Histone modification (*MLL*, *MLL2, MLL4*) and chromatin remodeling (*ATRX*, *DAXX*) were altered in 22% of tumors in the TCGA study (33, 34).

These data suggest that the Wnt/β-catenin, cell-cycle regulators (CDKs), TERT, histone modification, and chromatin remodeling are the commonly dysregulated pathways in ACC. Our group demonstrated that high CDK1 expression in ACC was associated with adverse clinical features and shorter overall survival. We showed *in vitro* and *in vivo* efficacy of the synergistic combination of multi-CDK inhibitor and a proteasome inhibitor in ACC (39).

### Methylome, Transcriptome, MicroRNA, and ACC Clustering

The ENSAT study incorporated the recursively partitioned mixture model (40) to show four different DNA methylation-based tumor clusters. Compared to benign adrenal cortical tumors, ACCs are globally more hypermethylated at the CpG islands in the promoter regions. Two of the clusters corresponded to previously described by the CpG Island Methylator Phenotype (CIMP) status based on their differential methylation profile. Consistent with several cancers such as gastric, ovarian, liver, and lung cancers, patients with hypermethylated ACC had a significantly shorter survival. The group that contained ACCs with differential hypermethylation was further divided into “CIMP-high” and “CIMP-low” subgroups. Patients with ACC in both subgroups had significantly shorter survival than that of the non-CIMP group, but those in the CIMP-high group had the shortest overall survival (41).

The clustering of mRNA expression profiles in the ENSAT study confirmed the existence of two main transcriptional clusters that are strongly correlated with survival. The C1A cluster displayed numerous pathogenic variants and DNA methylation alterations and is associated with poor outcomes. C1A group comprised largely of CIMP-high and CIMP-low, while almost all ACCs in the CIB group were non-CIMP. Interestingly, the C1B cluster displayed specific deregulation of two microRNA clusters and is associated with a good prognosis (33).

microRNA Illumina sequencing was performed on 45 ACCs and 3 normal adrenal gland samples in the ENSAT study. Confirming the previously reported data (42–44), *MIR483*, located on intron 2 of the *IGF2* locus, is overexpressed in ACC. Interestingly, a recent single-institution study of 48 patients suggested that mIR-483-5p measured after initial surgery for ACC can be a potential early post-operative biomarker for ACC prognosis to predict recurrence-free survival (45). In the ENSAT cohort, miRNA analysis revealed the upregulation of 11 miRNAs belonging to the miRNA-506-514 cluster (Xq27.3) and downregulation of 38 miRNAs to the imprinted *DLK1-MEG3* cluster (14q32.2). miRNA clustering in the ENSAT cohort showed three distinct clusters: Mi1, Mi2, and Mi3. Cluster Mi1 showed the largest miRNA expression differences relative to normal adrenal samples. Interestingly, the ACCs in Mi1 cluster had the downregulation of 38 miRNAs belonging to the imprinted *DLK1-MEG3* cluster (14q32.2). The SNP array identified the LOH of the 14q in all Mi1 tumors, with the associated transition from hemi to full-hypermethylated *MEG3* long noncoding RNA. Thus, the loss of the maternal unmethylated allele resulted in the silencing of the miRNA in the *DLK1-MEG3* cluster. Because the DLK1 is a non-canonical Notch ligand and was implicated in ACC tumorigenesis, targeting DLK1/Notch signaling may be further explored. Transcriptome clusters from the ENSAT group were strongly correlated with subgroups based on DNA methylation and miRNA expression. The C1A subgroup included almost all CIMP and Mi3 tumors, and the C1B subgroup was generally non-CIMP and belonged to Mi1 or Mi2 miRNA clusters. The mutation rate was noted to be higher in the C1A subgroup as compared to C1B (0.75 mutations/megabase vs 0.32 mutations/megabase; p = 7.5 × 10^-4^, Wilcoxon rank-sum test) (33).

Information from the clustering experiments described above contributed to further clustering experiments that were instrumental in formulating several molecular stratification systems. The clustering of 89 ACCs in the TCGA cohort based on their arm-level alterations produced three subgroups, named chromosomal (61%), noisy (30%), and quiet (9%). The chromosomal group had the highest frequency of whole-chromosome arm gains and losses, whereas the noisy group had a significantly higher number of chromosomal breaks, leading to worse overall survival. The quiet group had very few large copy-number alterations. Whole-genome doubling was seen in 68% of the noisy subtype, 51% of the chromosomal subtype, and 0% in the quiet subtype (34).

The TCGA study also performed clustering of ACC by genomic and transcriptomic characteristics that yielded a multitude of groups. A Cluster of Cluster (CoC) analysis was performed based on DNA copy-number, DNA methylation, mRNA-expression, and mi-RNA expression platforms. Three subtypes were delineated, named CoC I, II, and III. Disease progression rates were reported as 7%, 56%, and 96%, respectively. CoC I ACCs are characterized by low methylation of CIMP, steroid phenotype low pattern, and implicate the following genes: *ZNRF3*, *MEN1*, and MMR-related genes. CoC II ACCs have an intermediate level of methylation of CIMP, typically have steroid phenotype high pattern with/without proliferation pattern, and implicate the following genes: *CDKN2A*, *CTNNB1*, *NF1*, *PRKAR1A*, *TP53*, and *ZNRF3*. Lastly, CoC III ACCs show a high level of CIMP methylation, are steroid phenotype high with/without proliferation pattern, have the worst clinical outcomes, and have alterations in the following driver genes: *CDK4*, *CDKN2A*, *CTNNB1*, *MLL4*, *RB1*, *TERT*, *TP53*, and *ZNRF3* (34).

Another important study in understanding molecular markers of malignancy in ACC was performed by de Reyniès et al. (46). In their study, 153 unilateral adrenocortical tumors were analyzed by microarray or reverse transcription quantitative polymerase chain reaction. They discovered that among malignant tumors, the combined expression of *BUB1B* and *PINK1* was the best predictor of overall survival. *BUB1B* encodes a protein called BUBR1 that is important for proper chromosome separation during cell division by ensuring that each sister chromatid is attached to a spindle microtubule. Impaired checkpoint function has been implicated in cancer predisposition (47). *PINK1* encodes PTEN induced putative kinase 1, a protein located in the mitochondria and is thought to protect against malfunction during periods of cellular stress. Pathogenic variants in *PINK1* cause one form of Parkinson’s disease (48). High levels of *BUB1B-PINK1* is associated with a good overall prognosis and is classified as CoC I. On the contrary, low levels of *BUB1B-PINK1* are associated with a worse overall prognosis and are typically classified as either CoC II or III.

More recently, Drelon et al. performed a retrospective analysis of publicly available microarray data from Cochin and Michigan ACC cohorts (49). Tumors in these cohorts had overexpression of *EZH2*, and was further supported by mRNA sequence data from the TCGA program. *E2F* transcription factors are positive regulators of *EZH2* expression. The knockdown of specific E2F transcription factors resulted in a decrease of ACC cells *in vitro*, and can be considered as a target for future study.

## Recent Trials

ACC remains the neglected and lethal cancer because the progress in identifying clinically-effective therapy has been slow. Since the FIRM-ACT trial, no clinical trial has been successful to improve outcomes of patients with advanced ACC. Based on preclinical data above showing that 90% of ACC overexpressed *IGF2* and the inhibition of IGF2/IGF1R was effective *in vivo*, several clinical trials were conducted using monoclonal antibodies (cixutumumab and figitumumab) and a small molecule inhibitor (linsitinib). None has shown promising efficacy in ACC. The GALACCTIC trial (50) was a phase III, double-blinded, randomized controlled trial that evaluated patients with advanced ACC treated with at least one but fewer than three previous drug regimens. Patients were administered either linsitinib (an inhibitor of IGF1R and insulin receptors) or placebo. The trial failed to show a survival benefit compared to placebo (median OS, 323 days vs 356 days, p = 0.77). The resistance likely occurs because of the downstream events. Thus, additional therapy to address Wnt/β-catenin and overexpressed CDKs may be needed.

Similar to other solid cancers, immunotherapy such as checkpoint inhibitors have been studied in ACC. One of the main challenges in using immunotherapy in ACC is the concomitant Cushing’s syndrome that occurs in 50% of patients with ACC. Glucocorticoid excess causes T cell depletion and is associated with an unfavorable prognosis (51). The JAVELIN trial (52) was a phase Ib single-arm study that included 1,758 patients with different types of advanced solid tumors. Avelumab, an anti-PD-L1 antibody, was the treatment of interest. A subgroup analysis of 50 patients with advanced ACC previously treated with platinum-based chemotherapy showed a partial response in 6% of patients, two of whom were also treated with mitotane concomitantly. Other trials have also failed to show the benefit of streptozocin monotherapy (20), the combination of IGF1R and mTOR pathway inhibitors (3.9% response, n = 181) (53), tyrosine-kinase inhibitors (1.4% response rate, n = 72) (53), and gemcitabine-based chemotherapy (4.9% response rate, n = 145) (54).

## Bench to Bedside

Despite recent misses in ACC therapy development, there are many molecular targets currently being evaluated for the management of ACC. Each specific molecular dysregulation, whether it be from gene amplification, loss-of-function, or microsatellite instability, can potentially be treated with a specific targeted therapy. Because ACC is resistant to drug treatments for several reasons such as the overexpression of p-glycoprotein that effectively pumps the drugs out of the cells and multiple dysregulated pathways involved in tumorigenesis and cell survival, single-drug treatment is not likely to result in a durable long-term efficacy. The ideal treatments should target multiple critical dysregulated molecular features in ACC to induce a synergistic response tailored to patient’s tumor profile.

Our group demonstrated that the overexpression of *CDK1* and *CDK2* in multiple independent cohorts and was associated with poor prognosis. We found that patients with ACC overexpressing *CDK1* and *CDK2* had significantly higher rates of larger tumors, metastasis, recurrent disease, and shorter overall survival (39). Moreover, *CDK4* amplification has a prevalence of approximately 19% in ACC (55). Treating the NCI-H295R ACC cell line and ACC primary cultures with a CDK4/6 inhibitor, palbociclib, induced a concentration-dependent decrease of cell viability in cell culture. Cell cycle analysis revealed an increase in the proportion of cells at G0/G1 phase, and palbociclib significantly decreased expression levels of cell cycle-related protein cyclin D1 (56). However, a different preclinical study evaluated the effects of palbociclib and ribociclib in NCI-H295R and SW-13 cell lines with different results. Treatment with palbociclib induced cell cycle arrest and senescence, similar to the previous experiment, in these two cell lines. Palbociclib induced apoptosis in the NCI-H295R cell line, but treatment with a different CDK4/6 inhibitor, ribociclib, did not. Neither drug-induced apoptosis in the SW-13 cell line (57). Thus, these two drugs may not be ideal candidates to test in a clinical trial. Palbociclib and ribociclib are currently used to treat HR-positive, HER2-negative breast cancer (58). Because there is an urgent need for effective systemic treatments in rare cancers such as ACC, traditional drug development is not feasible due to the prohibitive cost and time needed for developing a new therapy. We previously demonstrated that drug repurposing using quantitative high-throughput screening (qHTS) of a clinically approved drug library is an effective and efficient way to identify active drugs in rare cancers (59–61). However, the monotherapy using CDK inhibitors in cancer can induce the NF-kB signaling pathway to protect cells from lethal consequences (62). We showed that the combination of flavopiridol (multi-CDK inhibitor) and carfilzomib (proteasome inhibitor) was synergistically effective *in vitro* using NCI-H295R, SW13, and BD140A ACC cell lines, and *in vivo* using NCI-H295R human ACC xenografts *via* the reduction of XIAP (anti-apoptotic protein) (39). We are currently developing a clinical trial in patients with advanced ACC using the combination of multi-CDK and proteasome inhibitors.

Loss-of-function in *ATRX* has a prevalence of about 15% in ACC (55). Loss of *ATRX* in human cancer cells has been shown to prime cells for alternative lengthening of telomeres to achieve replicative mortality (63). Cells that undergo these changes are sensitive to serine/threonine-protein kinase ATR inhibitors (i.e. VE-821) or agents that produce double-strand breaks, such as radiation therapy (64). ATR inhibitors have also been shown to sensitize cells to topoisomerase inhibitors (65). A phase I study of ATR inhibitor M6620 in combination with topotecan (a topoisomerase 1 inhibitor) was performed for various advanced solid tumors. This combination is the first to use the ATR inhibitor-chemotherapy combination. The therapy was well-tolerated. Three of 5 patients with platinum-refractory small-cell lung cancer had a partial response or prolonged stable disease (66). However, no patients with adrenocortical carcinoma were included in this study. The combination of ATR inhibitor and topotecan should be studied in ACC with the loss of *ATRX*.

Loss-of-function mutations in *NF1* or *MAP2K1* have a reported prevalence of 12% in ACC (55). MEK inhibitors have been shown to be effective in a phase I study for *NF1*-driven inoperative plexiform neurofibromas (67), melanoma, and glioma (58). There is no clinical trial in ACC using inhibitors of the MAPK signaling pathway.

Loss-of-function mutations in *ATM*, *BRCA1*, or *BRCA2* are uncommon in ACC with a prevalence of approximately 4% (55). PARP inhibitors are currently approved for *BRCA1/BRCA2* mutation ovarian carcinoma (68). The efficacy of PARP inhibitor olaparib has also been observed in other cancers with a deficiency of the homologous DNA repair system, namely metastatic breast cancer and metastatic prostate cancer (69, 70).

Microsatellite instability or hypermutator phenotype is seen in about 4% of ACC tumors. Solid tumors classified as hypermutated or microsatellite instability-high (MSI-H) have shown encouraging response rates (40% of 10 patients with mismatch repair-deficient colorectal cancers, 71% of 7 patients with mismatch repair-deficient non-colorectal cancers) to anti-PD-1 antibody (71). A recent phase II clinical trial studying pembrolizumab in advanced ACC showed an objective response rate of 14% (14 patients at 27 weeks evaluated; 2 patients had a partial response, 7 patients had stable disease, and 5 patients had progressive disease), though 13/14 tumor specimens were microsatellite-stable (72).

Loss-of-function mutations in *PTCH-1* have a prevalence of 2% in ACC. Vismodegib is a Smoothened inhibitor that is currently approved for basal cell carcinoma, and other *PTCH-1* mutated tumors have responded to this therapy in a recent phase II trial (73). The MyPathway trial is currently ongoing, and enrolling patients with molecular testing demonstrating an activating mutation of *SMO* or loss-of-function mutation of *PTCH-1*.

*JAG1* amplification or *NOTCH1* loss-of-function has also been the topic of a recent trial. The prevalence of these two genetic alterations in ACC is currently unknown. *JAG1* overexpressed in ACC has been linked to increased cell proliferation (74). NOTCH inhibition resulted in inhibition of cell proliferation in a Y1 mouse ACC cell line (75). In a recent phase I trial, four patients with ACC (3 with *JAG1* amplification, 1 with *NOTCH1* truncation) received a NOTCH inhibitor; one patient had a partial response (76).

The most common disease-driving mechanisms of ACC, as described in a previous section, are the p53-Rb and Wnt/β-catenin signaling pathways. Unfortunately, these pathways still lack specific targeted therapy on clinical trials at this time, though preclinical work shows some promise (77).

## Other Treatments in ACC

Various additional therapies and strategies have been used in the treatment of ACC, including radiation therapy, radiofrequency ablation (RFA), and peptide receptor radionuclide therapy (PRRT). In preclinical studies (78, 79), it has been suggested that mitotane has possible radiosensitizing properties, and when given in combination with ionizing radiation, can promote neoplastic growth inhibition. However, a retrospective SEER database study identified 74 patients that received radiotherapy out of 530 patients diagnosed with ACC. In their propensity score analysis, they concluded that radiotherapy did not improve overall or cancer-specific survival in patients with ACC (80). Radiation therapy seems best utilized in palliation of symtpoms rather than as a treatment modality (81). RFA has been utilitized in the management strategy of unresectable ACC tumors, and is most useful in tumors <5 cm (82). However, the long-term impact is not known. RFA is also used in the treatment of small liver metastases (82). PRRT has also been studied in patients with ACC (83). In a prospective study of 19 patients with metastatic ACC, 8 patients displayed radiometabolic uptake of any-grade intensity, and 2 patients displayed strong uptake in multiple lesions. The two patients with strong uptake were treated with PRRT, and both obtained overall disease control lasting 4 and 12 months, respectively. These are at best case reports with limited data, and should be properly studied in a clinical trial.

## Current Clinical Trials

There are currently 12 ACC studies actively enrolling patients, as seen on clinicaltrials.gov (**Table 1**). Three trials pertain to immunotherapy with anti-PD-1 antibodies: two phase II trials evaluating nivolumab plus ipilimumab; one phase II trial evaluating pembrolizumab for rare and inoperable tumors. ADIUVO-2 is a phase III trial comparing cisplatin/etoposide/mitotane and mitotane monotherapy after initial resection for ACC. ACACIA is evaluating the efficacy of cisplatin/etoposide as compared to observation/mitotane after primary resection of localized ACC. Three trials are currently evaluating the efficacy of cabozantinib, an inhibitor of receptor tyrosine kinase. One trial is evaluating cabazitaxel for ACC progression after previous chemotherapy lines. A cancer peptide therapeutic vaccine, administered with nivolumab, is being evaluated. Patients with ACC that has metastasized to the peritoneum can be enrolled in a phase II trial evaluating surgical resection and HIPEC (cisplatin intraperitoneally, sodium thiosulfate intravenously during HIPEC).

**Table 1:** Clinical trials that are actively recruiting for adrenocortical carcinoma.

Clinical trial	Phase	Intervention(s)	Location	Design	Primary endpoint(s)
Cabazitaxel Activity in Patients With Advanced Adrenocortical-Carcinoma Progressing After Previous Chemotherapy Lines (CabACC)	II	Cabazitaxel	Azienda Ospedaliera Spedali Civili di Brescia (Brescia, Italy)	Prospective, non-randomized, multicenter, open label, single arm, phase II study in patients with advanced ACC	To assess clinical benefit after 4 months of cabazitaxel in patients with locally advanced or metastatic ACC who progressed after cytotoxic therapy
Cabozantinib in Advanced Adrenocortical Carcinoma (CaboACC)	II	Cabozantinib-s-malate	University Hospital Würzburg (Würzburg, Germany)	Prospective, non-randomized, open-label, single arm, single center phase II in patients with locally advanced or metastatic ACC refractory to standard treatment	Progression-free survival at 4 months
Adjuvant Chemotherapy vs. Observation/Mitotane After Primary Surgical Resection of Localized Adrenocortical Carcinoma (ACACIA)	III	Cisplatin plus Etoposide vs Observation or Mitotane	ASST Spedali Civili di Brescia (Brescia, Italy)	Prospective, randomized, open-label, stratified, nation-based multicenter, phase II trial for patients with Ki67≥ 10% after resection with curative intent	Comparison of recurrence-free survival in patients treated with adjuvant chemotherapy ± mitotane vs no treatment or adjuvant mitotane
A Novel Therapeutic Vaccine (EO2401) in Metastatic Adrenocortical Carcinoma, or Malignant Pheochromocytoma/Paraganglioma (Spencer)	I/II	Biological: EO2401	MD Anderson Cancer Center (Houston, TX, USA), 8 other sites	Multicenter, phase I/II, first-in-human study to assess safety, tolerability, immunogenicity, and efficacy of EO2401 in metastatic ACC	Adverse events assessment
Surgery and Heated Intraperitoneal Chemotherapy for Adrenocortical Carcinoma	II	Cisplatin, Sodium thiosulfate, Cytoreductive surgery	Columbia University Medical Center (New York, NY, USA)	Prospective, non-randomized, open-label phase II trial in patients with ACC that undergo successful debulking will then undergo HIPEC	Progression-free survival
Cabozantinib in Treating Patients With Locally Advanced or Metastatic Unresectable Adrenocortical Carcinoma	II	Cabozantinib	MD Anderson Cancer Center (Houston, TX, USA)	Prospective, non-randomized, open-label, single arm, single center phase II in patients with locally advanced or metastatic ACC refractory to standard treatment	Progression-free survival at 4 months
Mitotane With or Without Cisplatin and Etoposide After Surgery in Treating Participants With Stage I-III Adrenocortical Cancer With High Risk of Recurrence (ADIUVO-2)	III	Cisplatin, Etoposide, Mitotane	MD Anderson Cancer Center (Houston, TX, USA)	Prospective, randomized, open-label, parallel assignment in patients with stage I-III ACC with high risk of recurrence	Compare effect of adjuvant mitotane alone with adjuvant mitotane + etoposide/cisplatin on recurrence-free survival in high-risk ACC after initial resection
Nivolumab Combined With Ipilimumab for Patients With Advanced Rare Genitourinary Tumors	II	Nivolumab, Ipilimumab	Dana-Farber Cancer Institute (Boston, MA, USA), 5 other sites	Prospective, non-randomized, multicenter, open label, single arm, phase II study in patients with advanced rare genitourinary tumors	Objective response rate
Cabozantinib-S-Malate in Treating Younger Patients With Recurrent, Refractory, or Newly Diagnosed Sarcomas, Wilms Tumor, or Other Rare Tumors	II	Cabozantinib-S-malate	National Cancer Institute (Bethesda, MD), 143 other sites	Prospective, non-randomized, multicenter, open label, single arm, phase II study in children and young adults with refractory sarcomas, Wilms Tumor, and other rare tumors	Objective response rate in children and young adults
Pembrolizumab in Treating Patients With Rare Tumors That Cannot Be Removed By Surgery or Are Metastatic	II	Pembrolizumab	MD Anderson Cancer Center (Houston, TX, USA)	Prospective, non-randomized, single center, open label, single arm, phase II study in patients with rare tumors	Non-progression rate Incidence of adverse events
Nivolumab and Ipilimumab in Treating Patients With Rare Tumors	II	Nivolumab, Ipilimumab	National Cancer Institute (Bethesda, MD), 939 other sites	Prospective, non-randomized, multicenter, open label, single arm, phase II study in patients with rare tumors	Objective overall response rates

## Author Contributions

GA was tasked with outlining and writing the review article. NN was the principal investigator tasked with reviewing the article, making edits, and contributing ideas. All authors contributed to the article and approved the submitted version.

## Funding

The research activity in this manuscript was supported by the Intramural Research Program, National Cancer Institute, NIH (ZIA BC 011286).

## Conflict of Interest

The authors declare that the research was conducted in the absence of any commercial or financial relationships that could be construed as a potential conflict of interest.
